# LED Optrode with Integrated Temperature Sensing for Optogenetics

**DOI:** 10.3390/mi9090473

**Published:** 2018-09-17

**Authors:** S. Beatriz Goncalves, José M. Palha, Helena C. Fernandes, Márcio R. Souto, Sara Pimenta, Tao Dong, Zhaochu Yang, João F. Ribeiro, José H. Correia

**Affiliations:** 1 Institute of Applied Micro-Nano Science and Technology—IAMNST, Chongqing Key Laboratory of Colleges and Universities on Micro-Nano Systems Technology and Smart Transducing, Chongqing Engineering Laboratory for Detection, Control and Integrated System, National Research Base of Intelligent Manufacturing Service, Chongqing Technology and Business University, Nan’an District, Chongqing 400067, China; sgoncalves@dei.uminho.pt (S.B.G.); Tao.Dong@usn.no (T.D.); Zhaochu.Yang@usn.no (Z.Y.); 2 CMEMS-UMinho, Department of Industrial Electronics, University of Minho, Guimaraes 4800-058, Portugal; jose.palha@dei.uminho.pt (J.M.P.); a65352@alunos.uminho.pt (H.C.F.); a68554@alunos.uminho.pt (M.R.S.); sara.pimenta@dei.uminho.pt (S.P.); jribeiro@dei.uminho.pt (J.F.R.); 3 Institute for Microsystems-IMS, Faculty of Technology, Natural Sciences and Maritime Sciences, University of South-Eastern Norway (USN), Postboks 235, 3603 Kongsberg, Norway

**Keywords:** silicon neural probes, LED chip, thermoresistance, temperature monitoring, optogenetics

## Abstract

In optogenetic studies, the brain is exposed to high-power light sources and inadequate power density or exposure time can cause cell damage from overheating (typically temperature increasing of 2 ${}^{\circ }$C). In order to overcome overheating issues in optogenetics, this paper presents a neural tool capable of assessing tissue temperature over time, combined with the capability of electrical recording and optical stimulation. A silicon-based 8 mm long probe was manufactured to reach deep neural structures. The final proof-of-concept device comprises a double-sided function: on one side, an optrode with LED-based stimulation and platinum (Pt) recording points; and, on the opposite side, a Pt-based thin-film thermoresistance (RTD) for temperature assessing in the photostimulation site surroundings. Pt thin-films for tissue interface were chosen due to its biocompatibility and thermal linearity. A single-shaft probe is demonstrated for integration in a 3D probe array. A 3D probe array will reduce the distance between the thermal sensor and the heating source. Results show good recording and optical features, with average impedance magnitude of 371 kΩ, at 1 kHz, and optical power of 1.2 mW·mm${}^{-2}$ (at 470 nm), respectively. The manufactured RTD showed resolution of 0.2 ${}^{\circ }$C at 37 ${}^{\circ }$C (normal body temperature). Overall, the results show a device capable of meeting the requirements of a neural interface for recording/stimulating of neural activity and monitoring temperature profile of the photostimulation site surroundings, which suggests a promising tool for neuroscience research filed.

## 1. Introduction

The central nervous system is the part of the human body that is least understood, and there is a constant effort to develop novel and useful tools and techniques to increase knowledge about it. Advances in microtechnologies allowed the development of micrometer-size devices that promote the interface between biological neural tissue and physical and electronic components. These instruments, known as neural probes, are usually invasive and with multiple recording sites [1].

Optogenetics is a recent technology that combines genetics and optics to promote stimulation or inhibition in specific photosensitive cells of brain tissue when exposed to light [2]. Combined with optogenetics, neural probes are now capable of simultaneously performing electrophysiology studies and stimulation based on light pulses, with increased cell-type selectivity and millisecond-scale temporal precision [3]. An optogenetic implantable tool is known as optrode.

Optrode designs can be categorized based on its approach to deliver light to the tissue, i.e., as devices integrating customized optical fibers, waveguide systems or LEDs. Commercial optogenetics-compatible neural probes, like those available by Neuronexus or Cambridge Neurotech, integrate exclusively optical fibers as light sources. These approaches present various drawbacks discussed in a recent review [4], where LED probes stand out by overcoming coupling light losses and maximizing delivered light power due to the proximity to target cells. Nowadays, there are various LED-based penetrating optrodes reported in the literature [5,6,7,8,9,10]. In our work, the LED optrode distinguishes from those designs due to integration of a temperature monitoring system.

Design requirements to manufacture a relevant optrode have been reported [4,11]. One of these challenges consists of preventing cell damage from overheating processes in the stimulation focus area. Thus, it becomes crucial to assess thermal properties of optical sites under various conditions, avoiding inadequate light-power density or exposure time, which can cause overheating. Probes providing in situ heat monitoring can be particularly important in academic scenarios, where photostimulation protocols are frequently customized to each experiment and application.

The core body temperature maintains a near constant (37 ${}^{\circ }$C) over a broad range of environment temperatures. However, the human brain is quite sensitive to fluctuations in temperature [12]. The knowledge on brain temperature fluctuations is limited, and, therefore, there is no established threshold above which irreversible heat-induced brain injury occurs [13]. Haveman et al. reported microscopic damage in many brain areas (striatum, cortex, hippocampus and thalamus) when subjected to temperatures of 39 ${}^{\circ }$C [14]. Rises in temperature of approximately 2 ${}^{\circ }$C have been used as a threshold to prevent brain damage [15], corresponding nowadays to the regulatory limit recommended by the American Association of Medical Instrumentation (AAMI). Nevertheless, this temperature reference may vary based on different species, animal age and brain activity state [13,15].

By directly exposing light sources to tissue, LED-based optrodes could be easily affected by overheating, as a light emitter converts energy into heat. Although previous studies using LED-optrodes have measured rises of temperature in vivo below 1 ${}^{\circ }$C (using thermal cameras) [16,17], monitoring device temperature is crucial, since the lack of monitoring could cause damage of neural cells and greatly disturb brain functions. In this regard, McAlinden et al. [17] and, more recently, Dong et al. [18] measured the heating profile of LEDs using thermal cameras. In this paper, an approach to manufacture a thin-film thermoresistance (RTD) sensor on an LED-optrode body is presented, capable of monitoring the temperature on the stimulation surroundings, preventing temperature rises over 2 ${}^{\circ }$C.

An RTD is a temperature sensor that operates on the measurement principle that a material’s electrical resistance changes with temperature. RTDs have been used to add functionality in biodevices for blood flow [19], heart [20], and superficial [21] and deep [22,23,24,25] brain measurement applications. For high-performance thermal sensing coupled to an optrode, the proposed thermal sensor needs to meet the following main requirements: (1) Micrometer-size dimensions, so it can be integrated in the probe body. For this application, thin-film RTDs, which enable smaller dimensions, were used. Thin-film RTDs allow good time responses, vibration resistance, and are relatively inexpensive and stable [26]; (2) Good resolution. RTD must be capable of monitoring temperature fluctuations in the medium that are inferior to the maximum increase in temperature before cell damage (2 ${}^{\circ }$C); (3) Temperature range of 0 ${}^{\circ }$C to 60 ${}^{\circ }$C. The wide temperature range was chosen for future applications, e.g., low temperatures required in neurosurgery procedures [27].

In this paper, a Pt RTD was fabricated using microfabrication lithographic methods. Pt RTDs were previously reported in gas [28] and heat [29] flow devices. Pt was chosen due to its biocompatibility and linear behavior with temperature variations within the proposed temperature range [30]. Moreover, Pt is the material also used for manufacturing the optrode recording sites, which avoids increasing fabrication complexity of the device.

In summary, the focus in this paper is to demonstrate a simple and robust manufacturing approach to produce a multifunction single-shaft probe for rodents’ applications, combining optogenetics with electrophysiology and temperature sensing, avoiding overheating processes. An 8 mm deep and 600 μm wide optrode coupled with a 300 μm long Pt RTD was successfully manufactured, capable of spanning nearly any mice brain structure. Electrochemical, optical and thermal characterization of the device is also presented and discussed, which validated the proposed device as a valuable tool in neuroscience.

## 2. Probe Design

As a device capable of delivering light to neurons and electrically recording them, the proposed optrode comprises 10 recording points (50 × 50 μm${}^{2}$) around a single LED chip (ELC-470-37, Roithner LaserTechnik GmbH, Wien, Austria) with dimensions of 280 × 310 × 85 μm${}^{3}$. The recording points are metallic Pt thin-films responsible to convert ionic into electronic currents, and therefore record electrical activity of neurons. The LED chip is the light source, which delivers light to photosensitive engineered brain cells, so they can express the intended biological effect.

**Figure 1:**
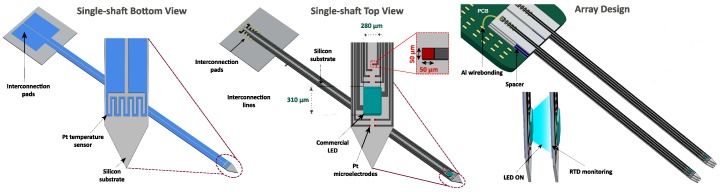
Design of the 3D silicon neural array concept. Pt thermoresistance (RTD) patterning on a single shaft (**bottom view**), and on the opposite side 10 recording sites and an LED chip (**top view**).

The proposed device also includes a Pt RTD for temperature sensing, aiming to prevent tissue overheating around the implant (>2 ${}^{\circ }$C). RTD is positioned in the shaft on the opposite site of the LED and recording sites, which would allow the temperature sensor to be positioned even closer to the stimulation focus of a neighbor shaft. Thus, this design becomes interesting for a close-packed 3D array by assembled individual shafts on top of each other, as illustrated in Figure 1. The goal of this study is to demonstrate a multifunctional probe, thus just the fabrication of a single shaft is demonstrated. The single-shaft configuration could be assembled into an array by the stacking method reported by Chang et al. [31].

**Figure 2:**
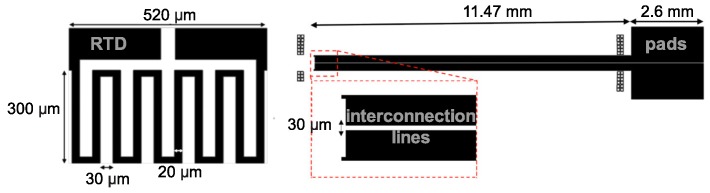
Design and geometrical dimensions of RTD patterned on the optrode.

RTD design must meet the dimension requirements of the proposed device. Therefore, its geometry was dimensioned based on Pouillet law—Equation (1)—that computes resistance (*R*) from input resistivity (ρ), length (*L*) and cross-sectional area (*t*, thickness and *W*, width) of the resistive material. To increase RTD’s length, a serpentine geometry (Figure 2) and the following parameters were chosen: *t* = 50 nm; *W* = 20 μm; theoretical $\rho_{Pt}$ = 1.05 × 10${}^{-4}$ Ω·mm; *L*= 3.27 mm, which resulted in an RTD area of 300 × 520 μm${}^{2}$, and a theoretical resistance of 343.35 Ω. The higher the length, the higher RTD resistance. Higher resistance can improve accuracy in one side but can also increase device noise. Thus, a sensible trade-off between those factors must be achieved. Top RTD geometry included a large area to promote a better electrical contact between the serpentine and its pads via interconnection lines. Pad resistance represents less than 2% of the RTD resistance:

(1) $$R\;=\;\rho \frac{L}{t\;W}.$$

The Si probe outline is 8 mm long and 600 μm wide with a sharp tip that facilitates probe implantation. Probe geometry is accomplished by conventional blade cutting technology, using a diamond blade (NBC-ZB 2050, Disco, Tokyo, Japan) suitable for Si wafer dicing [32].

## 3. Methods

This section includes the fabrication methodology and electrochemical, optical and thermal characterization processes used to manufacture and validate single-shaft optrodes.

### 3.1. Microfabrication

**Figure 3:**
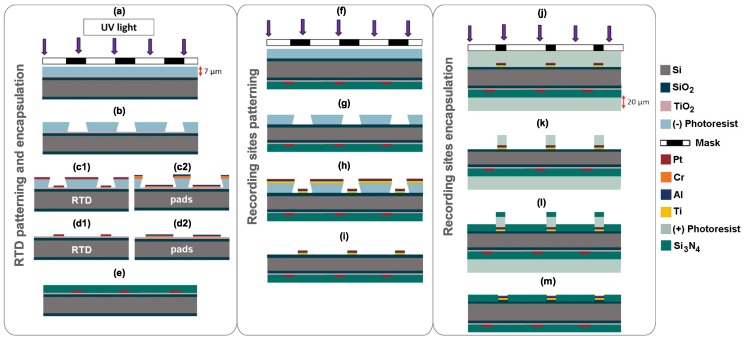
Cross-section view of the neural device fabrication process flow (not to scale).

Figure 3 summarizes the manufacturing process of the proposed single-shaft device. This process begins with the fabrication of the Pt RTD (Figure 1 Bottom view) followed by the manufacturing of the recording sites and the pads for the LED (Figure 1 Top view). This order aims to start with the simpler fabrication steps first.

In this paper, n-type [100] 525 μm thick Si wafers (with 1 μm of SiO${}_{2}$ at wafer surface) were selected for producing neural shafts. Si wafers were chosen due to the legacy of microfabrication technologies used for micromachining Si devices, their compatibility to complementary metal-oxide-semiconductor (CMOS) processes, and good mechanical proprieties [33]. The chosen Si doping and crystal orientation ensures the maximum shaft robustness after the dicing step. Initially, Si samples were cleaned with acetone on a 20 min ultrasonic bath, rinsed with deionized (DI) water and heated at 110 ${}^{\circ }$C during 20 min for dehydration. The cleaning step promotes a better adhesion of the substrate surface in the further fabrication steps.

**Figure 4:**
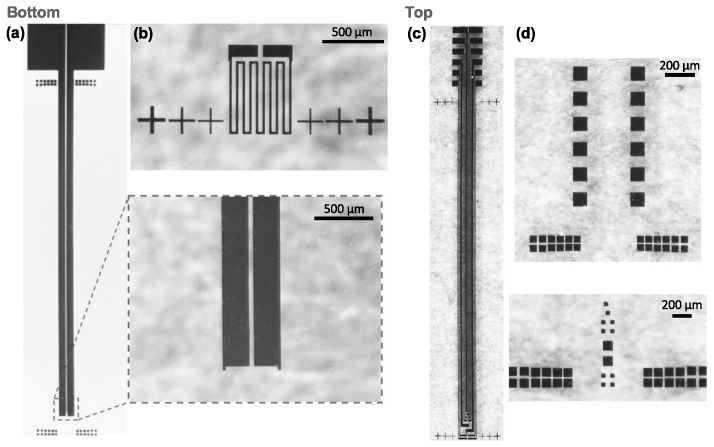
Lithographic masks used during fabrication process of the optrode. (**a**) RTD’s interconnection lines and pads; (**b**) RTD; (**c**) interconnection lines, recording sites, and pads for LED and recording points; (**d**) connection pads to external electronics (top) and exposure of recording sites and pads for the LED (bottom).

RTD is patterned by photolithography. Firstly, 10 nm of TiO${}_{2}$ as electrical insulation layer is deposited over the entire wafer to enhance adhesion between SiO${}_{2}$ surface of wafers and RTD and pad’s material [34]. Thin-film deposition parameters are shown in Table 1. Then, spin-coating of a 7 μm thick layer of negative photoresist (AZ nLOF 2070, MicroChemicals GmbH, Ulm, Germany) that is an image reversal resist. The samples are exposed to ultraviolet (UV) light (Figure 3a), using the lithographic mask in Figure 4b, and immersed in developer (AZ 726 MIF, MicroChemicals GmbH) to dissolve the unexposed photoresist (Figure 3b). Next, it is performed a metalization step (50 nm Pt) over the samples (Figure 3(c1)), to create RTD geometry—see Table 1. Then, the negative photoresist layer is lifted by its immersion in stripper (TechniStrip NI555, MicroChemicals GmbH), so that only the thin metal films remain in the substrate (Figure 3(d1)). After RTD patterning, steps (a) to (d) are repeated for interconnection lines and pads fabrication of the RTD using chromium and aluminum (30 nm Cr/600 nm Al) metallic layers (Figure 3(c2,d2))—see Table 1. For these steps, it used the mask in Figure 4a. Finally, an 800 nm Si${}_{3}$N${}_{4}$ passivation layer is deposited, thick enough to protect RTD and its pads (Figure 3e).

After RTD manufacturing, samples undergo lithographic steps (Figure 3f,g), using the lithographic mask shown in Figure 4c, with the same negative photoresist for the interconnection lines, pads and recording sites patterning. Then, Ti/Al/Pt (15 nm/200 nm/60 nm) metalization layers (Figure 3h) are deposited. Deposition parameters are shown in Table 1. Next, samples are again immersed in stripper (TechniStrip NI555, MicroChemicals GmbH), removing photoresist from the wafer (Figure 3i).

Another photolithographic process is performed to protect the samples against silicon dust during the dicing phase, sequentially on top and then on the bottom surface (Figure 3j). In this stage, a layer of 20 μm thick positive photoresist (AZ 4562, MicroChemicals GmbH) is deposited by spin-coating. Then, samples are exposed to UV light, using the mask shown in Figure 4d. Before the developer step in the pattering process, the cutting phase is performed in order to get the desired probe geometry, carried on a DAD-2H/6T dicing machine (Disco, Tokyo, Japan) performing cuts 150 μm thick. The cutting step is performed before passivation step because mechanical cutting of wafers introduces Si dust over the samples. Thus, the resist layer serves as a debris protective layer. Probe outline is set as 8 mm long, 600 μm wide with a sharp tip. A detailed dicing step for probe shaping is reported elsewhere [35], and the tip sharpening process is accomplished by using an automatic cutting program of the dicing machine, which allows the user to set a target cut angle (in this case, 45${}^{\circ }$).

The samples are then cleaned with DI water, and the photoresist removed with developer (AZ 351B, MicroChemicals GmbH), exposing only passivation area (Figure 3k). A deposition of 400 nm thick layer of Si${}_{3}$N${}_{4}$ as the insulation material was performed (Figure 3l), followed by the removal of the resist layer with acetone (top and bottom), exposing the recording sites and LED pads (Figure 3m). Finally, the blue-light LED chip is welded with solder paste (EM907, Kester) on the probe. LED’s contact pads are coated with a thin layer of a biocompatible transparent glue (PERMABOND 102), in order to protect LED against wet conditions.

After manufacturing, the optrode is fixed to a Printed Circuit Board (PCB) using cyanoacrylate, and its contact pads are packaged by Al wire-bonding. The PCB provides connection for external hardware for the LED chip and the RTD pads, and it is also coupled to an 18-pin connector (A79014-001, Omnetics, Minneapolis, MN, USA) to ensure external connectivity for recording sites.

### 3.2. Characterization

The characterization process of the proposed device aimed to validate its threefold goal: record electrical neural activity; stimulate engineered target cells sensitive to blue light; and monitor temperature profile around the probe. For this purpose, electrochemical, optical and thermal measurements were performed in vitro.

Electrochemical impedance spectroscopy (EIS) is a valuable technique in assessing the recording capabilities of recording sites and, because the voltage excursions at the electrode are small, may also be a useful and benign method for the in vivo assessment of an electrode [36]. The impedance measurements were performed in a Gamry system (Reference 600, Gamry Instruments, Warminster, PA, USA), using a standard three-electrode configuration: 40 × 40 × 0.25 mm${}^{3}$ Pt foil as counter electrode, Ag/AgCl as reference electrode, and 0.9% NaCl solution as electrolyte at room temperature. Impedance (Z) was measured for frequencies from 100 Hz to 1 MHz at a constant 10 mV rms alternating current (AC) voltage.

Photostimulation is validated by measuring power intensity of the light source. Reported minimum light intensity to promote a biological effect in engineered cells is 1 mW·mm${}^{-2}$ [3]. LED light power was measured using a photodiode sensor (FDS100-CAL, Thorlabs, Newton, NJ, USA), coupled to a 1 mm diameter pinhole. Power (*P*) can be obtained by Equation (2), where I is the current produced by the photodiode and ℜ is the photodiode’s responsivity at a wavelength (λ):

(2) $$P_{\lambda }\;=\;\frac{I}{{ℜ}_{\lambda }}.$$

The fabricated RTD was validated by measuring its resistance (*R*) with a four-wire setup. Temperature measurements were carried out inside a temperature-controlled furnace (0 ${}^{\circ }$C to 100 ${}^{\circ }$C and 5 ${}^{\circ }$C steps) coupled to an acquisition system (DT800, dataTaker, Scoresby, Australia) and software interface (DeLogger, dataTaker). A commercial RTD sensor, hereafter refereed as Pt100 (DM-510, Thorlabs), is used as comparative tool for the temperature measurements with a 600 μm long RTD. All measurements were carried out with a current of 0.1 mA. RTD’s temperature in ${}^{\circ }$C (*T*) can be obtained with its resistance (*R*), temperature coefficient of resistance (*TCR*) and resistance at 0 ${}^{\circ }$C (*R*${}_{0}$), as shows Equation (3) [29]. *TCR* is given by *R*${}_{0}$ and *R*${}_{100}$ (resistance at 100 ${}^{\circ }$C)—Equation (4) [37]:

(3) $$T\;=\;(\frac{R}{R_{0}}-1)TCR,$$

(4) $$TCR\;=\;\frac{R_{100}-R_{0}}{100\times R_{0}}.$$

RTD’s resistivity ($\rho_{exp}$) was obtained with van der Pauw method [38]. $\rho_{exp}$ can be obtained with Equations (5)–(7). Moreover, the sensitivity of the RTDs can be obtained as the slope of the second-order polynomial fit [39]:

(5) $$R_{A}=\frac{V_{12}}{2I_{43}}+\frac{V_{43}}{2I_{12}}\;\mathrm{and}\;R_{B}=\frac{V_{14}}{2I_{23}}+\frac{V_{23}}{2I_{14}},$$

(6) $$e^{\frac{-\pi R_{A}}{R_{S}}}+e^{\frac{-\pi R_{B}}{R_{S}}}\;=\;1,$$

(7) $$\rho_{exp}\;=\;R_{s}t.$$

## 4. Results and Discussion

The fabrication methodology based on lithography, thin-film depositions and blade dicing successfully accomplished an optrode design with the proposed features: 10 recording sites for electrical recording of neural activity; integration of one commercial LED chip for optical stimulation; and, finally, an RTD for temperature sensing of photostimulation site surroundings.

**Figure 5:**
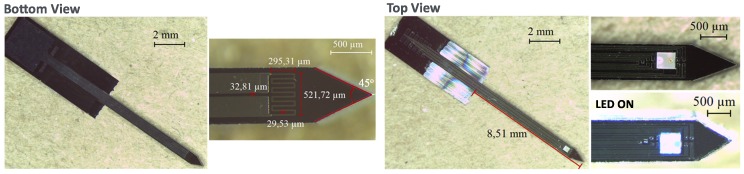
Results of the fabricated optrode integrating 10 Pt recording sites and commercial LED chip, and also a Pt RTD on its backside.

Microfabrication results are shown in Figure 5. Geometrical features of Si optrodes resulted in 8 mm long, 600 μm wide and 525 μm thick shafts. Maximize length of penetrating interfaces is important so the device is capable of reaching deeper neural structures than current designs [5]. For rodents’ applications, the probe cross-section must still be optimized. Here, it was demonstrated a single LED-based probe concept, whose dimensions are mainly limited by the dimensions of the commercial LED chip.

Traditionally, μ-LEDs are either (1) monolithical manufactured onto the device structure by deposition of gallium nitride (GaN) layers on a substrate [5,6]; or (2) integrated in the probe by LED transfer techniques [7,8,9,10]. Here, the latter approach due to employment of a commercial LED chip was used. While the first approach has the disadvantage of offering limited substrate choices, manual assembly of LED to substrate represents a harder task and might yield challenges. Further developments to our probe could include monolithically manufacture LEDs onto the probe, as demonstrated by other studies [5,40], ultimately leading to probe cross-section reduction. An interesting approach to address high-footprint commercial LED chips is reported by Ayub et al. [41]. In that study, LED chips are mounted on a thin polyimide-based substrate, stiffened using a micromachined ladder-like silicon structure. This approach avoids thicker probes by transfer LED chip to the surface of a stiff and thick substrate. Although minimizing probes cross-section is a preferable feature, with our approach, wider probes are necessary to accommodate wide LED chips and recording sites.

**Figure 6:**
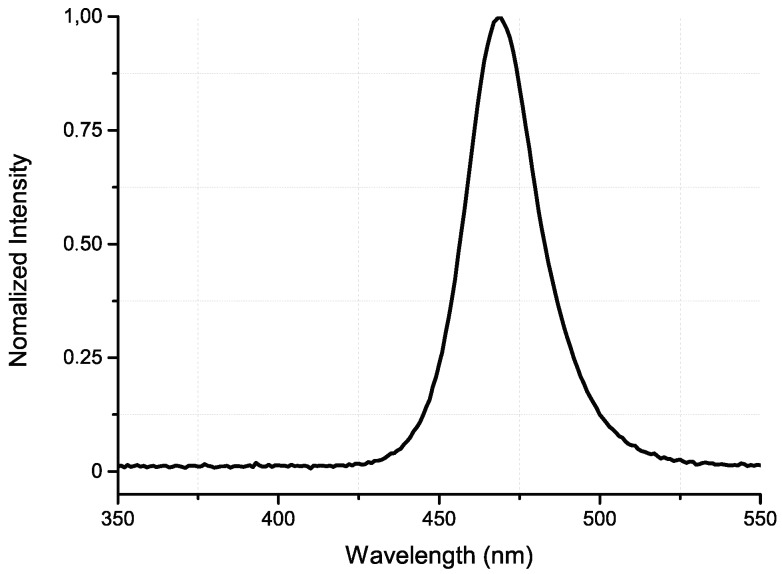
Experimental LED’s normalized light intensity as a function of the wavelength. LED peak intensity is at approximately 470 nm.

Light intensity tests for the LED chip, performed with the previously mentioned photodiode and pinhole, measured an average photodiode current of 168.5 μA when a current of 20 mA is applied to the LED. Considering the LED’s peak emission wavelength (approximately 470 nm—Figure 6) and the photodiode responsivity of 0.14 A/W (at 470 nm), extracted from its datasheet, LED optical power measured was 1.2 mW·mm${}^{-2}$—Equation (2). This result is superior to the reported minimum light intensity (1 mW·mm${}^{-2}$) to effectively promote photomodulation in brain tissue [3].

By using a thermal camera, McAlinden et al. [17] measured the temperature rise profile of 40 μm-diameter GaN LEDS. They reported a maximum temperature rise of 1.5 ${}^{\circ }$C over 100 ms light pulse. More recently, Dong et al. [18] demonstrated temperature variation over pulsed and continuous illumination regime, using the same forward current (20 mA) and a similar area (240 × 320 μm${}^{2}$) LEDs as the emitter proposed in this paper (250 × 280 μm${}^{2}$). Their results show a maximum temperature rise of 2 ${}^{\circ }$C for 350 ms pulse light train and 3 ${}^{\circ }$C for continuous irradiance over 15 min. Moreover, this study measured a 400 μm penetration depth (depth that can be attained while still presenting the optical power of 1 mW·mm${}^{-2}$) for a Lambertian emitter.

Another important geometrical characteristic of the probe is its tip shape. Here, Si shafts present sharpened tips (opening angle 45${}^{\circ }$). Sharp tips on these devices have been reported to result in lower implantation forces, and thus lower tissue damage [42,43,44].

Currently, a high-density probe includes more than 1000 channels [45,46,47], which advantageously span wider tissue areas and allow unprecedented opportunities for extracellular electrophysiology studies. On the other hand, they suffer higher signal attenuations by noise and crosstalk wiring. Conversely to these high-density designs, the proposed approach includes more functionalities (optical stimulation and temperature monitoring), not only recording capability as those reports. In fact, Kim et al. demonstrated a multi-functional operation that includes only a single 400 μm${}^{2}$ Pt recording site [22].

**Figure 7:**
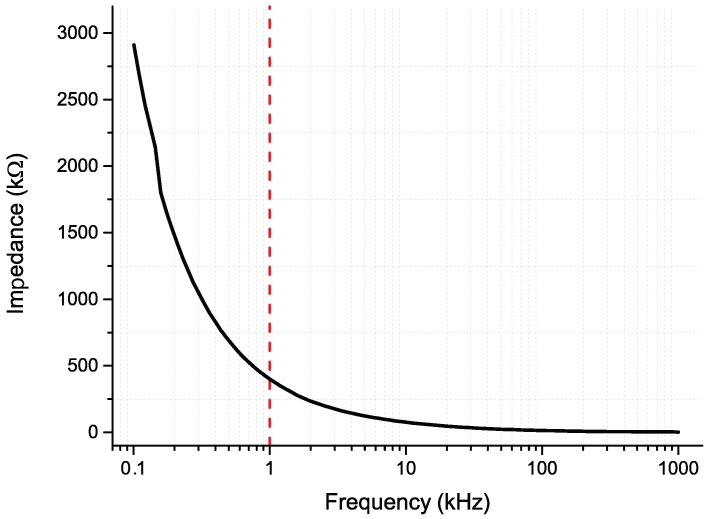
Impedance results for the Pt 50 × 50 μm${}^{2}$ recording sites.

Figure 7 shows EIS average result for the fabricated 50 × 50 μm${}^{2}$ recording sites. At 1 kHz (neurons firing rate), they show an average of 371 kΩ suitable for electrophysiology studies [48].

RTD was also successfully manufactured on one surface of the device. RTD design includes its location on the opposite side of the LED, which still makes it possible to monitor vicinity of the stimulation focus. In contrast to our approach, RTD could be fabricated next to the LED chip [24]. The downside of this approach is that it takes additional surface space in the shafts and overall complexity of fabrication to integrate an additional sensor. In this sense, Dehkhoda et al. reported an interesting study by presenting a temperature monitoring system that uses the LED both as emitter and its own sensor, taking advantage of the LED reverse current to measure the generated heat at the surface of the device.

**Figure 8:**
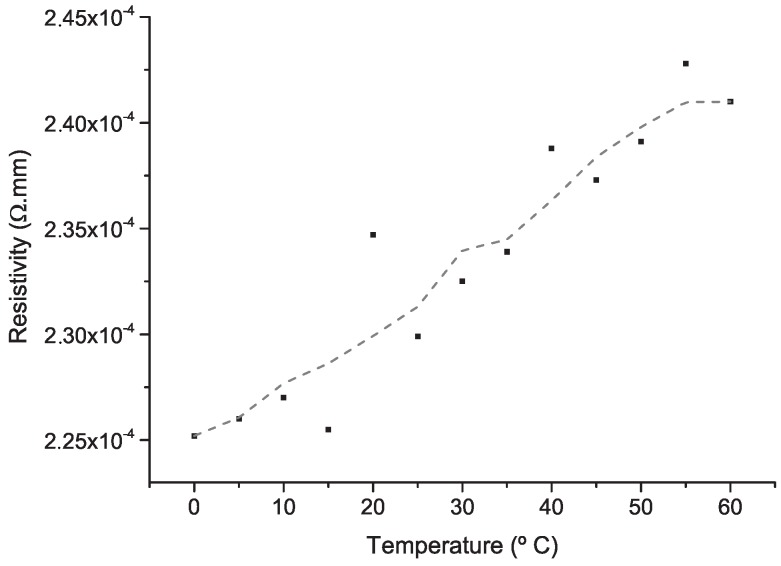
RTD’s resistivity vs. temperature. The dashed line results from a processing data five-point adjacent-averaging smoothing method, which replaces a point using the average of its five closest points.

Experimental Pt resistivity over the temperature range defined in the requirements (0 ${}^{\circ }$C to 60 ${}^{\circ }$C) is shown in Figure 8, where higher temperatures result in higher values of resistivity, as expected. Average RTD resistivity was 2.33 × 10${}^{-4}$ Ω·mm, similar to theoretical value (1.05 × 10${}^{-4}$ Ω·mm). RTD’s resistance at 0 ${}^{\circ }$C and 100 ${}^{\circ }$C, *R*${}_{0}$ and *R*${}_{100}$, respectively, were also measured to obtain the *TCR* coefficient of the fabricated RTD (Equation (4)). Table 2 shows the resistance values for RTD and Pt100. Pt100 *TCR* magnitude is consistent with the theoretical value of bulk pure platinum (0.0039 ${}^{\circ }$C${}^{-1}$) [49]. RTD’s sensitivity is 2.4 Ω·${}^{\circ }$C${}^{-1}$ in the temperature range of 35 ${}^{\circ }$C to 40 ${}^{\circ }$C. This value is in accordance with Pt RTDs reported by Fiedler et al., where Pt1000 and Pt5000 sensitivities were 1.7 Ω·${}^{\circ }$C${}^{-1}$ and 8.8 Ω·${}^{\circ }$C${}^{-1}$, respectively [50]. Table 3 compares the sensitivity and *TCR* values of the RTD in this work and previously reported studies.

**Figure 9:**
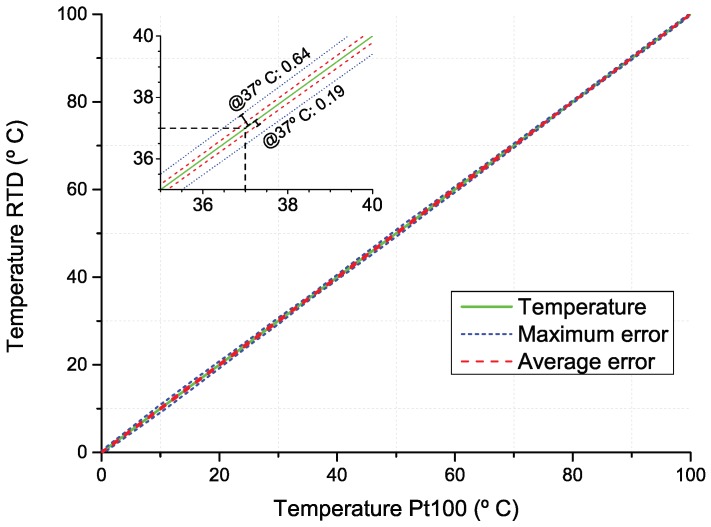
Comparative temperature measurements using Pt100 vs. RTD (green line). Measurement accuracy is given by error lines: maximum error (blue dashed line) and average error (red dashed line).

**Figure 10:**
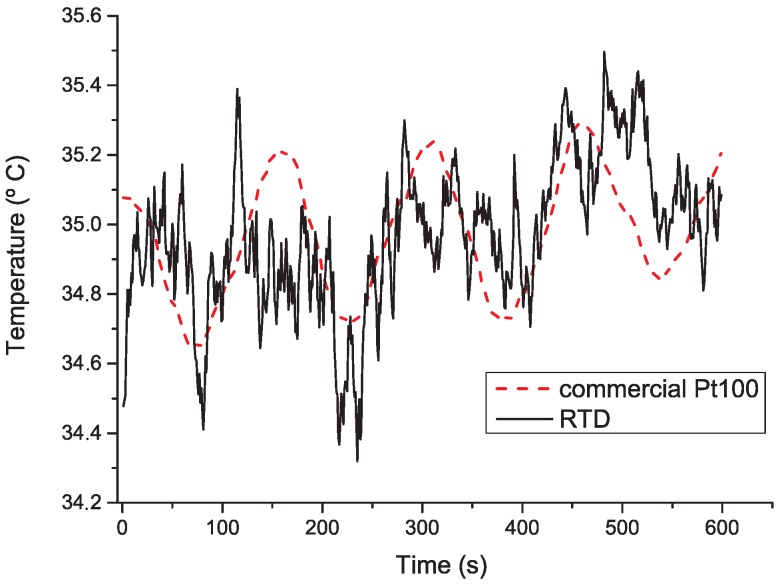
Measurements temperature results with a commercial Pt100 and the proposed RTD when medium is set to 35 ${}^{\circ }$C.

Figure 9 presents temperature measurements with Pt100 and RTD over a wide range of temperatures (0 ${}^{\circ }$C to 100 ${}^{\circ }$C), and at an approximately normal body temperature (35 ${}^{\circ }$C)—Figure 10. These results show RTD’s accurate temperature measurements in the entire range of temperatures. In addition, it is noticeable that RTD measurements show higher noise amplitudes relative to the Pt100 results, which might be related with higher thermal mass of the Pt100. In particular, at 37 ${}^{\circ }$C (normal body temperature), RTD has an average and maximum error of 0.19 ${}^{\circ }$C and 0.64 ${}^{\circ }$C, respectively. This means that temperature recording with the fabricated RTD might provide on average an estimated difference of 0.19 ${}^{\circ }$C from real tissue temperature. These results are suitable for monitoring temperature variations below 2 ${}^{\circ }$C required in this application. In Table 3, it is possible to see the final resolution is better than most RTD reported. In fact, even the RTD maximum error (0.64 ${}^{\circ }$C) presented is lower than most approaches reported to monitor brain thermal variation. Therefore, we believe a average error of 0.19 ${}^{\circ }$C is a promising result for this kind of devices.

Passivation layer on RTD is a required step with a twofold goal: (1) electrical insulation, and (2) avoiding electrical stimulation of neurons in its vicinity. Current as low as 10 μA has been reported to promote microstimulation of neurons as far as four millimeters away [54]. One possible limiting factor in RTD performance (response time) is the use of Si${}_{3}$N${}_{4}$ as a passivation layer due to low thermal conductivity. Fekete et al. demonstrated, however, a good thermal monitoring in mice tissue using a thin-film Pt sensor insulated with a Si${}_{3}$N${}_{4}$ layer [24].

Future work for this optrode-RTD combination design will include initially in vitro measurements of the environment thermal profile with the LED on, followed by in vivo validation of thermal brain monitoring in the vicinity of LED-based stimulation and electrophysiology studies.

## 5. Conclusions

The fabrication and in vitro validation of a single LED optrode was demonstrated in this paper. Its design accommodates optical stimulation, electrophysiological recording sites and temperature sensing with an RTD thin-film integrated in a silicon probe. The proposed multi-functional device is envisioned to help validated neural probes with optical stimulation capability, avoiding overheating processes. The manufacturing methodology relied on standard microfabrication technologies: lithography, thin-film depositions and low-cost traditional mechanical blade dicing technology. Fabrication results suggest a robust probe design, with 8 mm long single-shaft with a sharp tip. The 2D dicing methodology, applied to silicon wafers, facilitates the integration with patterning process, frequently used in MEMS and CMOS industry. Low impedance values of recording sites and sufficient light power results show great potential for this design to modulate neural activity in both cortical and deeper brain regions. RTD’s average accuracy of 0.2 ${}^{\circ }$C suggests that this is a promising tool for thermal mapping of brain tissue in the vicinity of the stimulation focus.

## Figures and Tables

**Table 1:** Parameters of the thin-films deposition to manufacture the optrode with RTD.

Material	Technology	Thickness (nm)	Pressure (mbar)	Gas injection (sccm)	Power (W)	Rate (Å/s)
TiO${}_{2}$	RF sputtering	10	2 × 10${}^{-3}$	10 (Ar); 2 (O${}_{2}$)	200	0.1
Pt	DC sputtering	50 and 60	6 × 10${}^{-3}$	40 (Ar)	100	3.4
Cr	e-beam	30	6.3 × 10${}^{-6}$	–	140	1
Al	e-beam	600 and 200	5.3 × 10${}^{-6}$	–	700	23
Ti	e-beam	15	4.3 × 10${}^{-6}$	–	350	0.8
Si${}_{3}$N${}_{4}$	RF sputtering	800 and 400	6 × 10${}^{-3}$	7 (Ar); 13 (N${}_{2}$)	150	0.3

**Table 2:** Resistance values at 0 ${}^{\circ }$C (*R*${}_{0}$) and 100 ${}^{\circ }$C (*R*${}_{100}$) for RTD and commercial Pt100. The calculated *TCR* value is also included.

Sample	*R* ${}_{0}$	*R* ${}_{100}$	*TCR*
Pt100	100.23 Ω	137.71 Ω	0.0037 ${}^{\circ }$C${}^{-1}$
RTD	1548.58 Ω	1787.55 Ω	0.0015 ${}^{\circ }$C${}^{-1}$

**Table 3:** Comparison of RTD developed in this work and previous studies.

Ref.	Material	Sensitivity (Ω·${}^{\circ }$C${}^{-1}$)	*TCR* (${}^{\circ }$C${}^{-1}$)	Resolution (${}^{\circ }$C)
[19]	Au	-	-	0.03
[20]	Poly-Si	-	-	0.9
[49]	Pt	0.781	0.0028	-
[50]	Pt	8.8	-	0.5
[51]	Pt	-	0.0015	1
[52]	Au	-	0.0032	0.25
[53]	Pt	1.485	0.0035	-
This work	Pt	2.4	0.0015	0.19

## Abbreviations

The following abbreviations are used in this manuscript:

RTD	Resistance Temperature Detector
e-beam	Electron-Beam
EIS	Electrochemical Impedance Spectroscopy
UV	Ultraviolet
DI	Deionized
*TCR*	Temperature Coefficient of Resistance
CMOS	Complementary Metal-Oxide-Semiconductor
RF	Radio-Frequency
DC	Direct Current
AC	Alternating Current
